# Immune exhaustion in ME/CFS and long COVID

**DOI:** 10.1172/jci.insight.183810

**Published:** 2024-10-22

**Authors:** Natalie Eaton-Fitch, Penny Rudd, Teagan Er, Livia Hool, Lara Herrero, Sonya Marshall-Gradisnik

**Affiliations:** 1 National Centre for Neuroimmunology and Emerging Diseases,; 2 Consortium Health International for Myalgic Encephalomyelitis, and; 3 Institute for Biomedicine and Glycomics, Griffith University, Gold Coast, Australia.; 4 School of Human Sciences (Physiology), The University of Western Australia, Perth, Australia.; 5 Victor Chang Cardiac Institute, Australia.

**Keywords:** Immunology, Adaptive immunity, Innate immunity

## Abstract

Myalgic encephalomyelitis/chronic fatigue syndrome (ME/CFS) and long COVID are debilitating multisystemic conditions sharing similarities in immune dysregulation and cellular signaling pathways contributing to the pathophysiology. In this study, immune exhaustion gene expression was investigated in participants with ME/CFS or long COVID concurrently. RNA was extracted from peripheral blood mononuclear cells isolated from participants with ME/CFS (*n* = 14), participants with long COVID (*n* = 15), and healthy controls (*n* = 18). Participants with ME/CFS were included according to Canadian Consensus Criteria. Participants with long COVID were eligible according to the case definition for “Post COVID-19 Condition” published by the World Health Organization. RNA was analyzed using the NanoString nCounter Immune Exhaustion gene expression panel. Differential gene expression analysis in ME/CFS revealed downregulated IFN signaling and immunoglobulin genes, and this suggested a state of immune suppression. Pathway analysis implicated dysregulated macrophage activation, cytokine production, and immunodeficiency signaling. Long COVID samples exhibited dysregulated expression of genes regarding antigen presentation, cytokine signaling, and immune activation. Differentially expressed genes were associated with antigen presentation, B cell development, macrophage activation, and cytokine signaling. This investigation elucidates the intricate role of both adaptive and innate immune dysregulation underlying ME/CFS and long COVID, emphasizing the potential importance of immune exhaustion in disease progression.

## Abstract 2

Investigation of immune exhaustion in chronic fatigue syndrome and long COVID using transcriptomics.

## Introduction

Myalgic encephalomyelitis/chronic fatigue syndrome (ME/CFS) and long COVID are 2 debilitating chronic illnesses that have garnered attention for their complex and overlapping clinical presentation (1). Both illnesses are characterized by postexertional malaise, severe fatigue, and cognitive dysfunction with significant effects on quality of life (QoL). The global prevalence of ME/CFS is estimated to be between 0.4% and 2.5%, and it is believed that the prevalence is likely to increase following the COVID-19 pandemic, while the prevalence of long COVID is estimated to affect between 5% and 43% of SARS-CoV-2 infections (2, 3). These illnesses are further burdened by the absence of a validated biomarker to differentiate between ME/CFS and long COVID to provide diagnostic assistance. Both ME/CFS and long COVID pose significant public health concerns with great economic effects.

Several of the hypothesized mechanisms for ME/CFS and long COVID pathogenesis share commonality including immune dysregulation, neuroinflammation, microbiota dysbiosis, and impaired energy production (4). In conjunction with the overlapping symptomatology, there is much debate on whether ME/CFS and long COVID are different conditions or the same. Immune dysregulation is a key feature of the pathogenesis of ME/CFS and long COVID (5). The immune system maintains homeostasis and defends the host against pathogens and other insults. However, prolonged exposure to antigens, inflammatory mediators, and cellular stressors can result in a phenomenon known as immune exhaustion. Immune exhaustion is characterized by the progressive loss of effector functions and increased expression of inhibitory receptors on immune cells (6, 7). This is exemplified by the upregulation of checkpoint molecules such as programmed cell death 1 (PD-1), cytotoxic T lymphocyte antigen 4 (CTLA-4), and T cell immunoglobulin and mucin domain protein 3 (TIM-3) on the surface of T lymphocytes and NKG24 on NK lymphocytes. These checkpoint molecules work to dampen immune responses and promote immune tolerance.

In long COVID, dysregulated immune responses are characterized by persistent inflammation, elevated proinflammatory cytokines, lymphopenia, and dysfunctional T cell responses (3). Specifically, immune profiling in long COVID reported elevated human leukocyte antigen (HLA), major histocompatibility complex class II (MHC-II) expression cells, increased exhausted CD4^+^ and CD8^+^ T cell subsets, reduced memory T cell numbers, and cytokine alteration (8). In ME/CFS, studies have reported altered cytokine profiles, abnormal T cell function, and impaired NK cell cytotoxicity (9–12). Previous research employing flow cytometry has found elevated exhausted T cell phenotype (CD4^+^PD-1^+^) in ME/CFS (13, 14). *HLA* alleles were found to be associated with the pathogenesis of ME/CFS (15), while single nucleotide polymorphisms (SNPs) in *CTLA4* were associated with postinfectious onset (16). To the authors’ knowledge, there have been no investigations into immune exhaustion using concurrent ME/CFS and long COVID cohorts.

Understanding the commonalities and differences in immune disturbances across ME/CFS and long COVID is critical to elucidate the pathogenesis behind these conditions. The identification of overlapping immune abnormalities provides valuable insights that may inform future diagnostic and therapeutic approaches. Immune exhaustion, a state of functional impairment and reduced responsiveness, has emerged as a potential contributing factor of both ME/CFS and long COVID. Therefore, this investigation aimed to elucidate transcriptome changes associated with immune exhaustion concurrently in patients with ME/CFS or long COVID.

## Results

### Participants.

This current study included *n* = 18 healthy controls (HC), *n* = 14 participants with ME/CFS, and *n* = 15 participants with long COVID. There were no significant differences between participant cohorts for age, sex, or highest level of education achieved (Table 1). Body mass index (BMI) differed significantly between cohorts (adjusted *P* value [*P*_adj_] = 0.021), whereby HC reported a significantly lower BMI compared with participants with long COVID (*P* = 0.016). Full blood count was determined for all participants. The number of monocytes (*P* = 0.049) and basophils (*P* = 0.030) differed significantly between groups. The number of monocytes was significantly higher in HC and participants with long COVID compared with those with ME/CFS; however, significance was lost following Bonferroni corrections for multiple comparisons. Basophils were significantly higher in HC compared with those with ME/CFS; however, significance was lost following Bonferroni corrections for multiple comparisons. All QoL variables differed significantly between cohorts. For all 36-item short-form health survey (SF-36) domains, participants with ME/CFS or long COVID reported significantly lower QoL compared with HC. For all World Health Organization (WHO) Disability Assessment Schedule (DAS) domains, participants with ME/CFS or long COVID reported significantly higher levels of disability compared with HC. No significant differences were reported between participants with ME/CFS and participants with long COVID, excluding the WHODAS Self-Care domain, in which patients with ME/CFS reported more difficulty (*P*_adj_ = 0.048). No participant with long COVID reported multiple SARS-CoV-2 infections at the time of sample collection. All participants reported any previous or current diagnoses. Reported comorbidities included fibromyalgia (*n* = 2) and postural orthostatic tachycardia syndrome (POTS, *n* = 3) in people with ME/CFS. A history of post–Epstein-Barr virus chronic fatigue was reported by *n* = 2 people with long COVID; however, reported chronic fatigue had resolved prior to SARS-CoV-2 infection. Irritable bowel syndrome (IBS) was reported by *n* = 2 people with long COVID and *n* = 2 people with ME/CFS. All clinical characteristics and demographics of participants are summarized in Table 1.

Participants with ME/CFS or long COVID were required to report on the symptom they regularly experience, with focus on the past 30 days’ frequency (how often they experienced the symptom), and severity (very mild to very severe) of symptoms. The occurrence of symptoms enabled the determination of case criteria fulfilled. All participants with ME/CFS met the Canadian Consensus Criteria (CCC), excluding 1 participant who reported an improvement in cognitive disturbances since a prior appointment with the Neuroimmunology and Emerging Diseases (NCNED) and fulfilled Fukuda criteria, thus demonstrating the fluctuating nature of the illness. All participants with long COVID fulfilled the WHO working case definition for “Post COVID-19 Condition.” There were no significant differences in the prevalence of symptoms between ME/CFS and participants with long COVID. The prevalence of symptoms is reported in Table 2.

### Differential gene expression.

**Figure 1:**
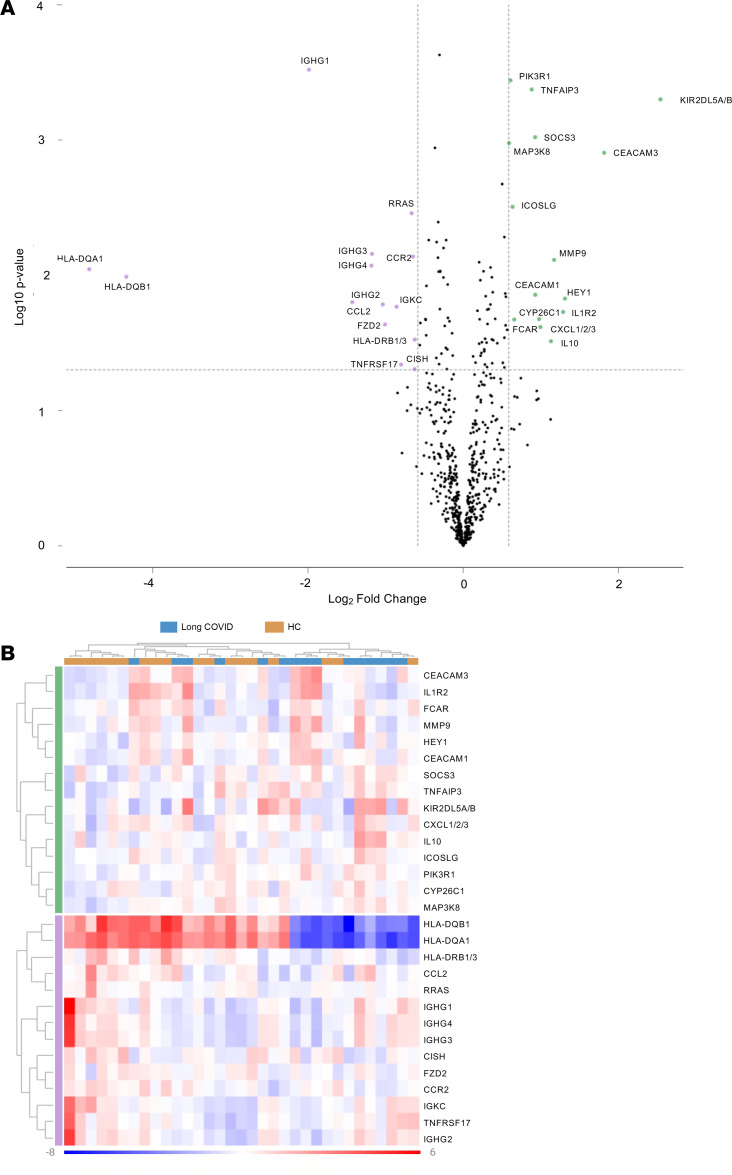
Differentially expressed genes in long COVID. (**A**) Volcano plot displaying statistical significance (log_10_[*P* value] on the *y* axis and log_2_ fold change on the *x* axis). Selected genes meeting filter criteria are presented as those downregulated (≤-1.5) and those upregulated (≥1.5). (**B**) Heatmap of selected genes representing log_2_ normalized expression values from –8 to 6. Red indicates high levels of expression, while blue indicates low levels of expression. Clusters are organized according to upregulated or downregulated genes by participant cohort. HC, healthy control.

Differential expression of genes was filtered according to log fold change (FC) parameters –1.5 to 1.5 and a *P* value of 0.05, resulting in the selection of 29 genes in long COVID compared with HC (Table 3 and Figure 1). Of the 29 selected genes, 15 were upregulated and 14 were downregulated. Downregulated genes, including *HLADQA1* and *HLADQB1*, had the highest degree of differential expression (log_2_FC = –4.81925, *P* = 0.009, and log_2_FC = –4.34154, *P* = 0.0102, respectively). Of the upregulated genes, *KIR2DL5A/B* had the highest degree of change with log_2_FC of 2.54102 (*P* = 0.0005).

**Figure 2:**
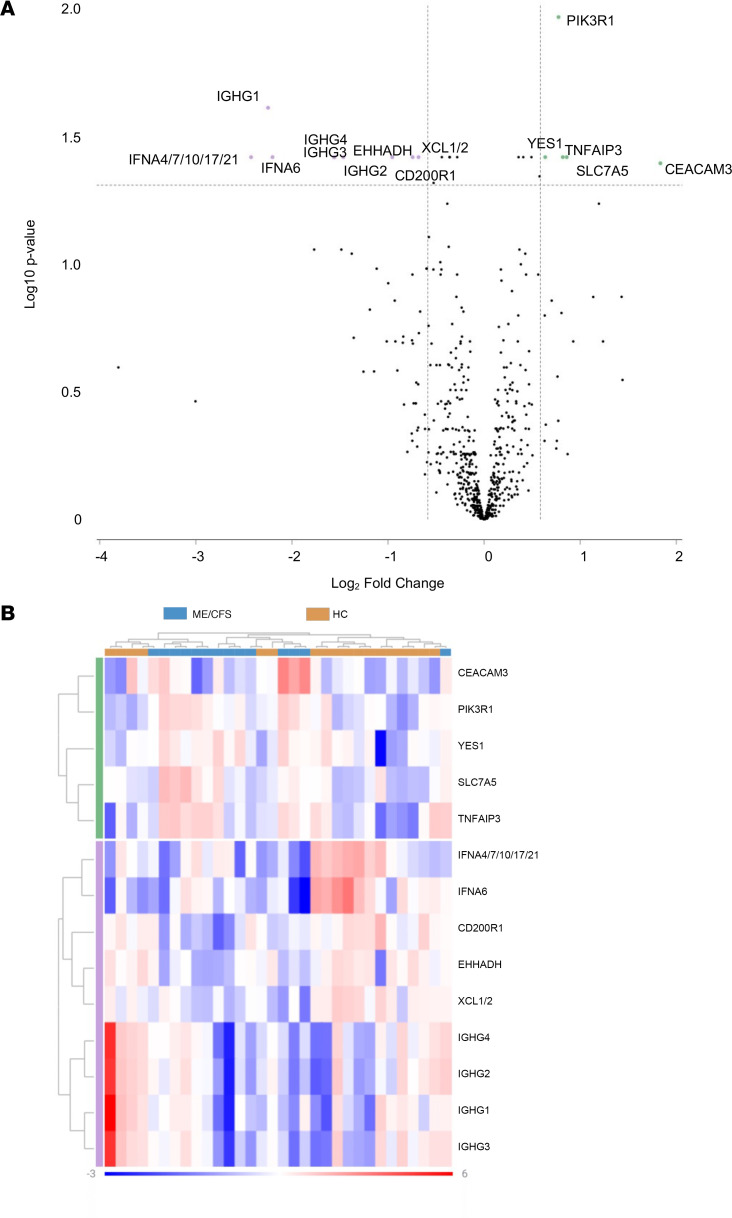
Differentially expressed genes between ME/CFS. (**A**) Volcano plot displaying statistical significance (log_10_[*P* value] on the *y* axis and log_2_ fold change on the *x* axis). Selected genes meeting filter criteria are presented as those downregulated (≤ –1.5) and those upregulated (≥1.5). (**B**) Heatmap of selected genes representing log_2_ normalized expression values from –3 to 6. Red indicates high levels of expression, while blue indicates low levels of expression. Clusters are organized according to upregulated or downregulated genes by participant cohort. HC, healthy control.

**Figure 3:**
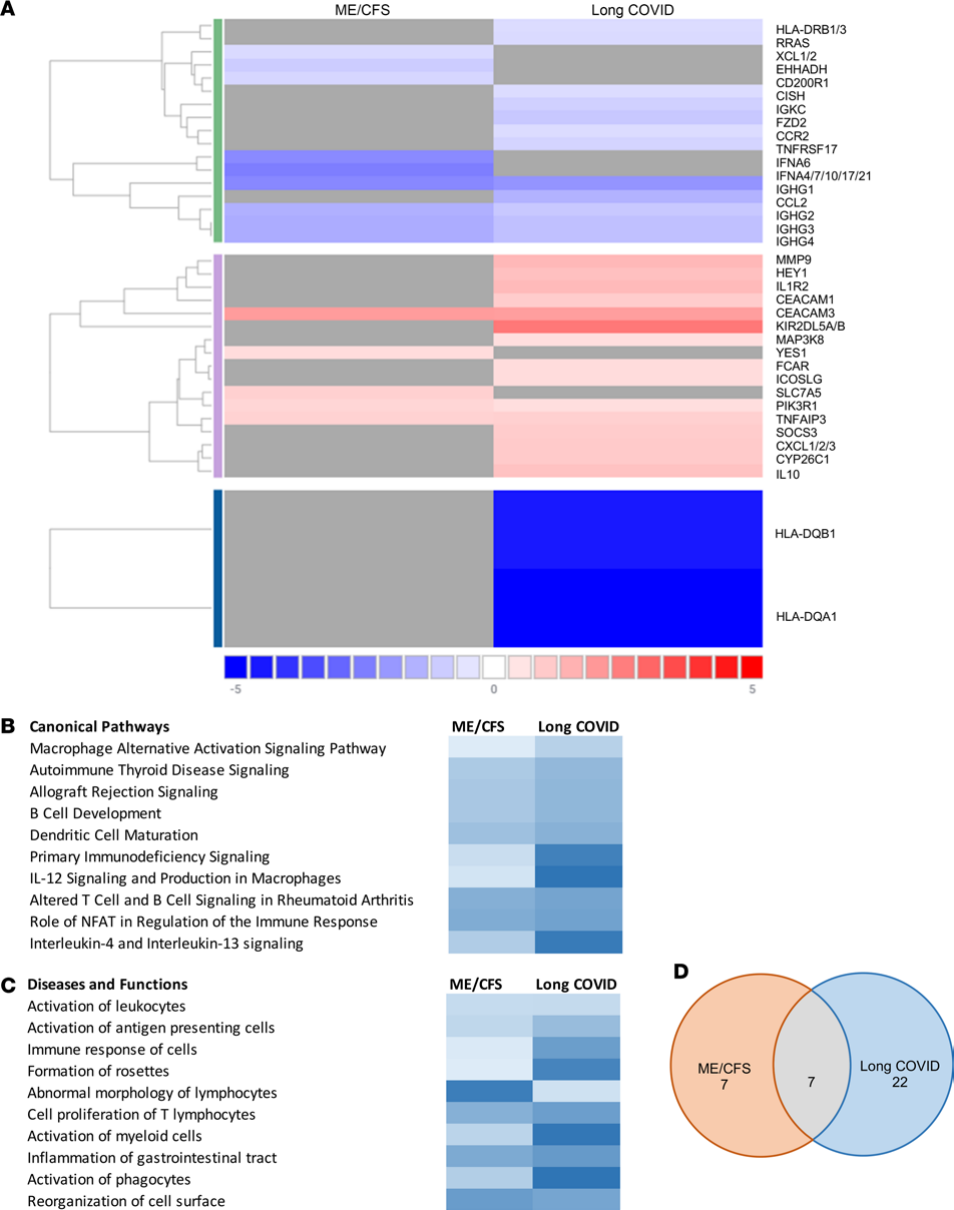
Overlapping gene expression in ME/CFS and long COVID. (**A**) Heatmap representing log_2_ normalized expression values (–5 to 5). Red represents higher expression, blue represents low expression, and gray represents no differential expression. Data exported from Rosalind Bio. (**B** and **C**) Heatmap of the top 10 canonical pathways (**B**) and diseases and functions (**C**). The darker gradient indicates greater significance. *P* < 0.05. Data exported from IPA. (**D**) Unique and overlapping genes. HC, healthy control.

A total of 14 genes was selected as differentially expressed between patients with ME/CFS compared with HC (Table 4 and Figure 2, A and B). Of the 14 genes, 5 genes were upregulated and 9 genes were downregulated. Downregulated genes, including *IFNA4/7/10/17/21*, *IGHG1*, and *IFNA6*, had the highest degree of differential expression (log_2_FC = –2.42502, *P* = 0.0005; log_2_FC = –2.24777, *P* = 0.000008; and log_2_FC = –2.20172, *P* = 0.0009, respectively). Of the upregulated genes, *CEACAM3* had the highest degree of change with a log_2_FC of 1.83403 (*P* = 0.0014). Hierarchical clustering grouped mRNA expression and samples according to similarity in expression patterns. Interpretation of the heatmap demonstrates similarities within ME/CFS and long COVID cohorts and similarities in expression patterns, except for a few samples (Figure 2A). Overlapping genes can be found in Figure 3, A and D. The full data set outputs can be found in Supplemental Data 2 (supplemental material available online with this article; https://doi.org/10.1172/jci.insight.183810DS1.)

### Gene set analysis.

The change in regulation within each gene set relative to the baseline was described using gene set analysis (GSA); both undirected enrichment score (UES) and directed enrichment score (DES) for the top 15 gene sets are presented in Table 5. Differentially expressed genes in long COVID were associated with PD-1 signaling (UES = 1.7782, DES = –1.742), IL-6 signaling (UES = 1.6638, DES = 0.9619), TGF-β signaling (UES = 1.6145, DES = 1.2819), antigen presentation (UES = 1.589, DES = –0.6682), mitogen activation protein kinase (MAPK) signaling (UES = 1.5361, DES = –0.4939), and mTOR signaling (UES = 1.4928, DES = 0.3127). In ME/CFS, GSA identified enrichment of gene sets including peroxisome proliferator–activated receptors (PPAR) signaling (UES = 1.9043, DES = –1.4736), fatty acid metabolism (UES = 1.7918, DES = –1.5557), NK receptors (UES = 1.7526, DES = –1.3492), glycolysis and glucose import (UES = 1.7244, DES = –1.5434), anergy (UES = 1.6924, DES = 1.4338), B cell exhaustion (UES = 1.6487, DES = –1.5374), and epigenetic modification (UES = 1.5496, DES = 0.9327).

Overlapping gene sets included chemokine signaling (long COVID: UES = 1.6212, DES = –1.4999; ME/CFS: UES = 1.9815, DES = –1.8727), type I IFN signaling (long COVID: UES = 1.5237, DES = –0.7452; ME/CFS: UES = 1.8554, DES = –1.5052), type II IFN (long COVID: UES = 1.5971, DES = 0.092; ME/CFS: UES = 1.7313, DES = –0.8088), TNF signaling (long COVID: UES = 1.6121, DES = 1.1078; ME/CFS: UES = 1.5776, DES = 0.7148), CTLA4 signaling (long COVID: UES = 1.5185, DES = –0.7578; ME/CFS: UES = 1.7201, DES = –1.1482), DAP12 signaling (long COVID: UES = 1.4937, DES = 1.115; ME/CFS: UES = 1.5637, DES = 1.1961), Janus kinase/signal transducers and activators of transcription (JAK/STAT) signaling (long COVID: UES = 1.594, DES = 0.978; ME/CFS: UES = 1.6042, DES = 0.3461), and other IL signaling (long COVID: UES = 1.5995, DES = –0.7734; ME/CFS: UES = 1.7592, DES = –1.078).

### Cell type abundance.

**Figure 4:**
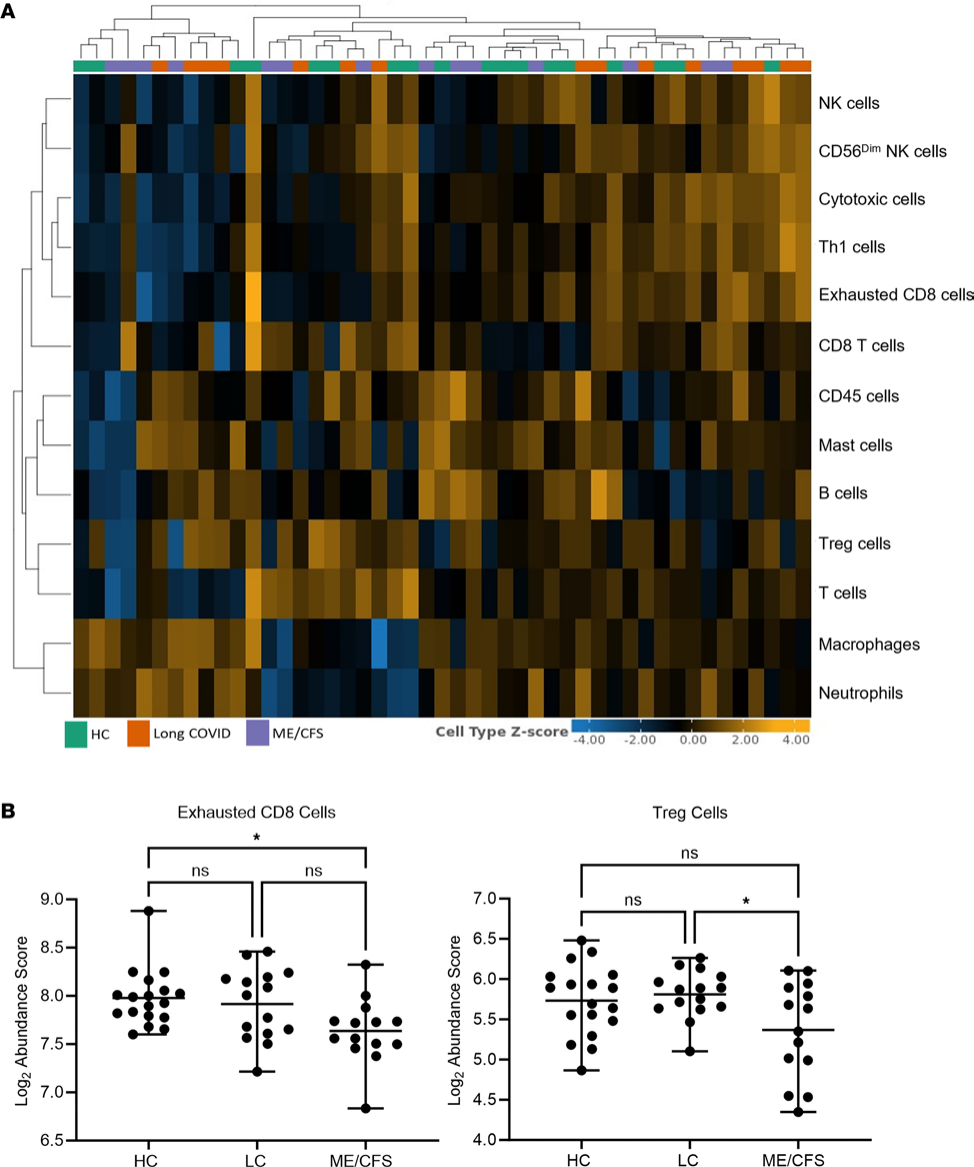
Cell profiles and gene expression. (**A**) Heatmap extracted from Rosalind Bio. Cell type *Z* scores for cell populations are populated for samples collected from ME/CFS, long COVID, and HC. (**B**) Comparison of cell type abundance scores extracted from Rosalind Bio; statistical analysis and the figure were completed using GraphPad Prism. Exhausted CD8 cells were compared using Kruskal-Wallis test with Dunn’s multiple-comparison corrections. Tregs were compared using 1-way ANOVA with Bonferroni’s multiple-comparison test. Graphs show mean with minimum and maximum ranges. Data are presented as mean with maximum and minimum range. *P < 0.05. HC, healthy control; LC, long COVID.

The abundance of cell populations was determined according to the expression of cell marker genes using Rosalind Bio. Hierarchical cluster analysis observations demonstrate heterogeneity within cohorts (Figure 4A). The abundance of exhausted CD8 cells was significantly lower in ME/CFS compared with HC (*P*_adj_ = 0.0147). There were no significant differences in normal CD8 T cells reported. Furthermore, the abundance of Tregs was significantly lower in ME/CFS compared with long COVID (*P*_adj_ = 0.0375) (Figure 4B). No other significance was reported; remaining abundance scores for cell types are shown in Supplemental Data 3.

### Pathways and disease functions.

Ingenuity Pathway Analysis (IPA) was used to determine the association of differentially expressed genes with biological functions and canonical pathways for both ME/CFS and long COVID cohorts when compared with HC (Table 6). The top 5 biological functions in long COVID include abnormal morphology of lymphocytes (*P* < 0.0001), activation of leukocytes (*P* < 0.0001), immediate hypersensitivity (*P* < 0.0001), activation of antigen-presenting cells (*P* < 0.0001), and lack of lymphocytes (*P* < 0.0001). Only 2 of the abovementioned biological functions overlap with the ME/CFS cohort: the activation of leukocytes (*P* < 0.0001) and the activation of antigen-presenting cells (*P* < 0.0001). The remaining top biological functions in ME/CFS include the formation of rosettes (*P* < 0.0001) (cell type not specified), immune response of cells (*P* < 0.0001), and activation of myeloid cells (*P* < 0.0001).

Top 5 canonical pathways differed between long COVID and ME/CFS, excluding the macrophage alternative activation signaling pathway (*P* < 0.0001 and *P* < 0.0001, respectively). Canonical pathways identified in long COVID included antigen presentation (*P* < 0.0001), autoimmune thyroid disease signaling (*P* < 0.0001), allograft rejection signaling (*P* < 0.0001), and B cell development (*P* < 0.0001). Meanwhile, IL-12 signaling and production in macrophages (*P* < 0.0001), primary immunodeficiency signaling (*P* < 0.0001), role of macrophages, fibroblasts and endothelial cells in rheumatoid arthritis (*P* < 0.0001), and neutrophil extracellular trap signaling pathways (*P* < 0.0001) were reported in ME/CFS. *HLA-DQA1*, *HLA-DQB1*, and *IGHG1* were found to be pivotal in the top biological pathways and diseases in long COVID, which overlapped with ME/CFS with the addition of *IGHG3*, *CCL2*, *CEACAM3*, and *IFNA6*. Overlapping pathways and disease function were observed in Figure 3, B and C. The complete pathways and disease function output can be found in Supplemental Data 4.

### Network analysis.

**Figure 5:**
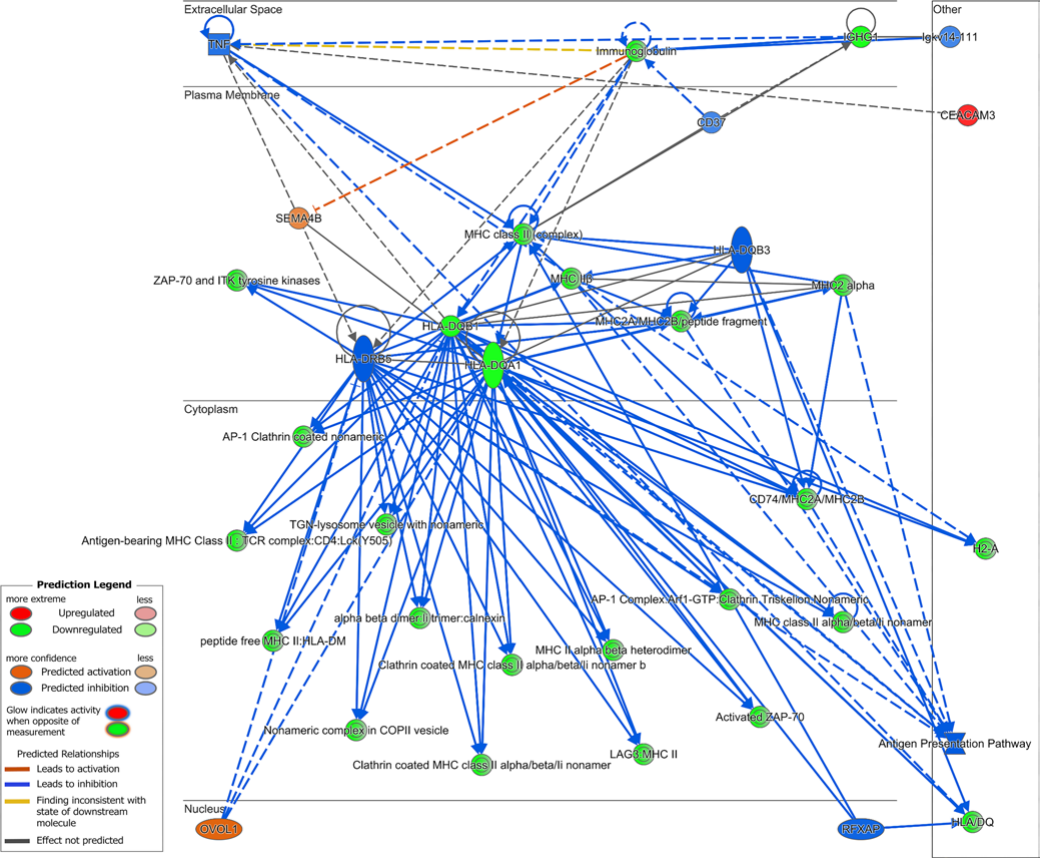
Network analysis in long COVID. Gene interaction network map consisting of top filtered differentially expressed genes. Genes are organized according to subcellular space. Network analysis score = 13.

**Figure 6:**
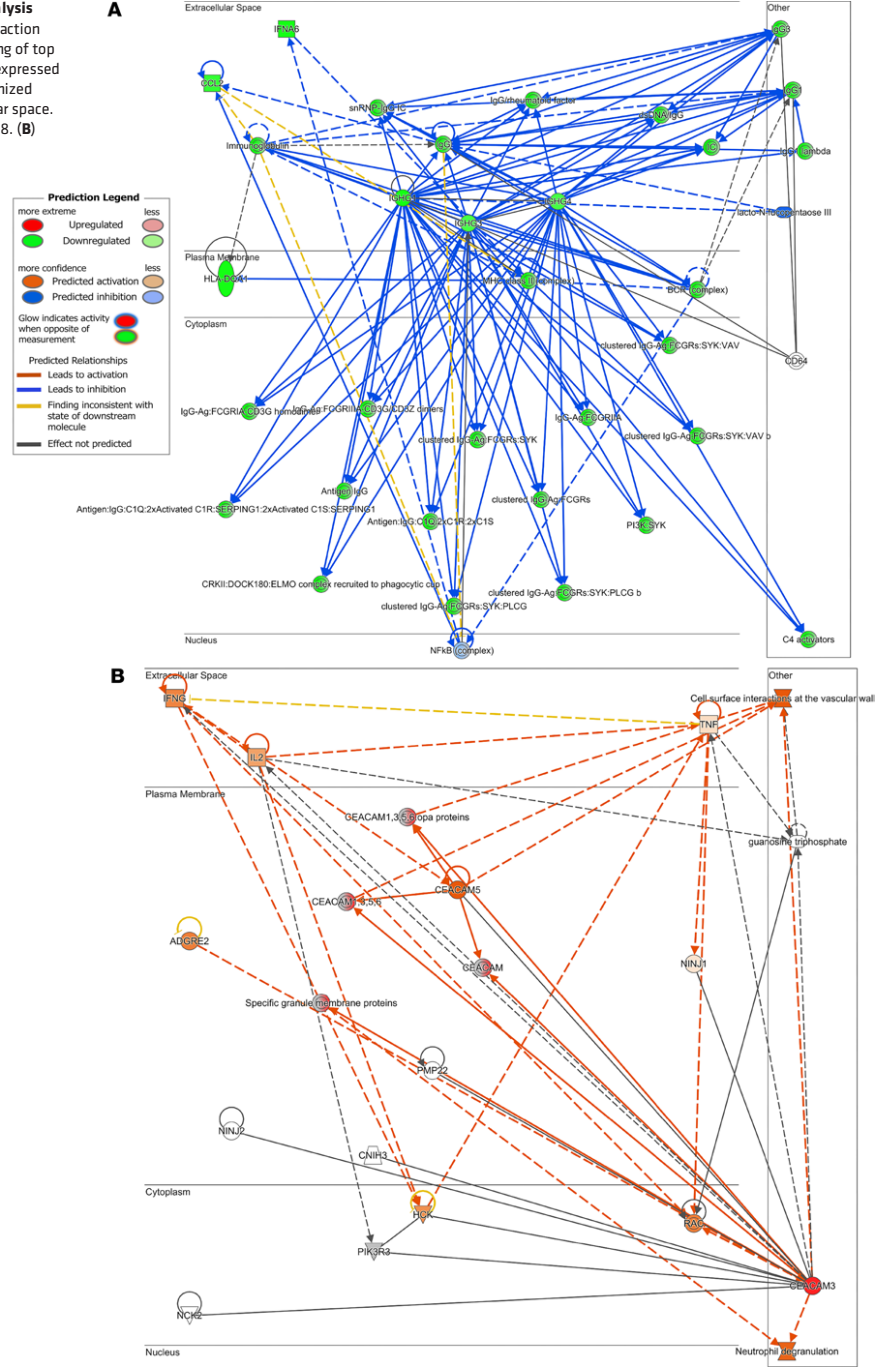
Network analysis in ME/CFS. Gene interaction network map consisting of top filtered differentially expressed genes. Genes are organized according to subcellular space. (**A**) Network 1 score = 18. (**B**) Network 2 score = 3.

Interaction network analysis was performed using IPA. This analysis demonstrates the interactions between molecules and the data set imported. One network was identified for long COVID (Figure 5), while 2 networks were identified for ME/CFS (Figure 6, A and B), of which network 1 (Figure 6A) obtained the highest score of 18. Network analysis in long COVID was associated with categories and disease or functions including gastrointestinal disease (chronic colitis, *P* < 0.0001), humoral immune response and protein synthesis (quantity of IgG1, *P* < 0.0001), immunological disease, injury or abnormalities (immediate hypersensitivity, *P* < 0.0001), and cell morphology and abnormalities (morphology of lymphocytes, *P* < 0.0001). Analysis for the highest scoring network in ME/CFS was associated with cellular assembly and organization (formation of rosettes, *P* < 0.0001), cellular development, hematological system development and function (maturation of Th cells, *P* < 0.0001), and cell-to-cell signaling and interactions (activation of antigen presenting cells, *P* < 0.0001). Network analysis outputs can be found in Supplemental Data 4.

## Discussion

This study investigates altered gene expression related to immune exhaustion in ME/CFS and long COVID concurrently. As an overview, 5 genes were significantly upregulated and 9 genes were significantly downregulated in people with ME/CFS compared with HC. In long COVID, 15 genes were significantly upregulated and 14 genes were significantly downregulated compared with HC. Seven differentially expressed genes overlapped between patient cohorts, suggesting the involvement of similar molecular pathways involved in immune dysfunction. A greater number of genes was identified, and 3 times the amount of genes was upregulated in long COVID compared with participants with ME/CFS.

The analysis revealed distinct patterns of gene expression and pathway dysregulation in both ME/CFS and long COVID cohorts. In ME/CFS, downregulation of IFN signaling (*IFNA4/7/10/17/21* and *IFNA6*) pathways and immunoglobulin genes (*IGHG*) suggests a state of immune suppression. However, this may differ from previous research findings reporting elevated *IFNA* (17–19) and upregulated *IGH* variable region genes (20), contradicting the role of autoimmunity in the pathogenesis of ME/CFS in this cohort. Notably, pathways related to macrophage activation and cytokine production were significantly affected, indicating dysregulation of innate responses. Regarding the occurrence of immune exhaustion in ME/CFS, downregulation of IFN-γ production is associated with T cell and NK cell exhaustion (7, 21–23), thus supporting the hypothesis on the role of immune exhaustion in ME/CFS. This is further supported by previous data on the expression of NKG2A, cytokine disturbances, and functional impairments. Cytokine dysregulation is commonly reported in ME/CFS; however, research findings are often contradictory (19). Increases in proinflammatory cytokines are reported in ME/CFS compared with HC, specifically TGF-β, TNF-α, and various IL types (24, 25). The results of the present study once again highlight the heterogeneity of ME/CFS and emphasize the importance of large sample cohorts to enable stratification on patient subsets.

Patients with long COVID exhibited dysregulated expression of genes involved in antigen presentation, cytokine signaling, and immune cell activation. Lingering elevated cytokine levels, otherwise known as the cytokine storm, in long COVID is consistently reported (26). A triad of IL-1β, IL-6, and TNF-α is associated with long COVID (27). In the present study, gene expression of *TNFAIP3* was significantly upregulated in both ME/CFS and long COVID, presenting potential consistency with previous research. Furthermore, both the upregulation (*KIR2DL5A/B*) and downregulation (*HLADQA1* and *HLADQB1*) of genes associated with antigen presentation suggest a heightened immune response, potentially reflecting persistent immune activation following viral infection. Biomarkers of exhaustion not commonly discussed also include the downregulation of *HLADQA1/B1* genes. Interestingly, the proportion of HLA-expressing cells was found to correlate with COVID-19 severity — specifically, a lower proportion of HLA-DR^+^ cells was reported in fatal infections (28). Conversely, a higher proportion of HLA-DR^+^ cells in severe to mild cases of COVID-19 was reported (29). Additionally, *HLADQB1* alleles are associated with chronic infection (30) and viral clearance (31). The expression of *HLADQB1* serves a vital role in effective immune responses against viruses, and downregulation may be attributed to virally induced immune avoidance or chronic immune activation.

Downregulated *IGHG* genes reported in ME/CFS were also observed in long COVID. Frequency and function of *IGHG* genes are associated with infectious immunodeficiency, allergy, autoimmunity, and malignancy. Interestingly, an increase in IGHG-expressing cells was detected during disease progression in the bronchoalveolar lavage fluid of patients with COVID-19 (32). IgG has a central role in primary immunodeficiency disorders (33). Low serum IgG levels and low levels of IgG subclasses are correlated with diminished defense against pathogens. Various bacterial and viral antigens and allergens affect individuals with particular *IGHG* haplotypes (33). The investigation of alternative *IGHG* genes and allele genotypes may elucidate their role in ME/CFS and long COVID. The decreased expression of *IGHG* may indicate immunodeficiency in participants with ME/CFS or long COVID and may explain their susceptibility to secondary or prolonged infections. Previous investigations have reported that individuals with IgG deficiency often complain of fatigue and that this deficiency is associated with lower QoL (34). However, IgG levels were not investigated in this current investigation.

To the authors’ knowledge, there is limited use of NanoString in research investigating ME/CFS and long COVID. Previous NanoString technology employed in ME/CFS reported protein kinase gene expression in NK cells (35). This publication reported on 37 upregulated and 55 downregulated genes associated with JAK/STAT and NF-κB activity in a cohort of participants with severe ME/CFS compared with HC. This aligns with results of the present study reporting significantly altered expression of *PI3KR1* in participants with ME/CFS or long COVID. However, other protein kinase genes included within the Immune Exhaustion panel did not significantly differ. It should be noted that the approach to analysis differed between current and previous research. Previous investigations of altered protein kinase expression or phosphorylation were associated with changes in overall immune cell function and linked with mediators of signaling cascades such as the regulation of calcium (35, 36).

GSA found similarities between participants with ME/CFS and long COVID, including chemokine signaling, type I and II IFN, IL signaling, CTLA4 signaling, DAP12 signaling, JAK/STAT signaling, and TNF signaling. Additionally, the identification of shared biological functions and canonical pathways between ME/CFS and long COVID, such as aberrant lymphocyte morphology and leukocyte activation, highlights commonalities in immune dysregulation across these conditions. This suggests potential overlapping mechanisms in disease pathogenesis and provides a rationale for exploring common screening and therapeutic strategies targeting immune exhaustion pathways. In ME/CFS, a complex interplay between immune regulation, metabolic pathways, and metabolism may be involved in pathogenesis (4, 5, 37). The role of metabolic pathways in ME/CFS has been investigated. The role of mitochondrial disturbances and the production of energy is well reported (38, 39). However, the mechanisms behind mitochondrial disturbances are elusive, and further research is required. It is possible that the role of mitochondria is greater in ME/CFS compared with long COVID. Furthermore, IL signaling, NK receptors, and JAK/STAT signaling highlight involvement of cytokine-mediated immune responses, and impaired signaling cascades align with the literature (12, 35, 36). Moreover, enrichment of gene sets related to B cell development and CTLA4 signaling in long COVID suggests altered adaptive immune responses and immune checkpoint regulation.

Gene expression according to cell type revealed the significance of exhausted CD8 cells and Tregs in ME/CFS. Suggested markers of immune exhaustion for CD8 T cells include the upregulation of CD38, CD39, TIM-3, HLA-DR, CTLA-4, NKG2A, CD107a, and PD-1 and the downregulation of IL-2, IL-21, IFN-λ, and granzyme B (40–43). The upregulation of immune checkpoints in this present study, including CTLA4 and PD-1, are typical features of exhausted CD8 T cells, and this upregulation suggests reduced effector T cells and impaired differentiation, proliferation, and function. A previous investigation by Gil et al. reported that participants with long COVID or ME/CFS have dysfunctional CD8^+^ T cells (44).

The significant differences in gene-associated cell abundance for Tregs in ME/CFS and long COVID is interesting. Previous investigations reported differences in Tregs in ME/CFS compared with HC, supporting an increase in Tregs perhaps as a method to control an overactive immune system (14, 45). Conversely, other investigations have reported a significant reduction in Tregs compared with HC (46). Studies have shown that patients with COVID-19 have considerably fewer Tregs compared with the general population. While the pathophysiology of COVID-19 appears to be influenced by Tregs, there is, however, limited research to comment on the role of Tregs in the development of long COVID (47, 48). Tregs are subsets of T cells that suppress the immune system. A decline in Tregs in the presence of chronic inflammation can result in further damage; conversely, diminished Tregs may be associated with immune exhaustion. This is supported by research suggesting that a combined Treg depletion and programmed cell death ligand 1 blockage results in immune exhaustion and autoimmune profiles. Tregs and PD-1 promote immune exhaustion, which minimizes the immunopathological collateral damage that occurs during a chronic viral infection (49), suggesting a potential effect in checkpoint signaling in people with ME/CFS compared with long COVID. This has consequences on immune regulatory and diminishing inflammatory inhibition resulting in continued tissue damage (50).

This current pilot investigation is not without limitations. Indeed, the small cohort sizes make it difficult to sufficiently stratify the heterogenous nature of ME/CFS and long COVID according to clinical presentation. This emphasizes the need for further investigations with larger cohorts to differentiate subtypes and identify biomarkers for patient stratification. However, in an attempt to control for potential confounding variables, codiagnoses of other chronic immune conditions were excluded. It is important to highlight that while this current investigation raised disease pathways associated with thyroid disease, rheumatoid arthritis, and allograft rejection, these conditions were excluded from the present study. This current research serves as the basis to justify further larger investigations to elucidate immunological biomarkers in these conditions. Furthermore, the Immune Exhaustion panel developed by NanoString biases expression analysis to a small selection of genes. While NanoString technology may provide sensitive, reliable, and reproducible results, future analysis may consider the validation of gene expression analysis using quantitative PCR.

This investigation reports transcriptome changes in participants with ME/CFS or long COVID concurrently using the NanoString Immune Exhaustion panel. Altered gene expression indicates that both innate and adaptive immune responses are indicated in the pathology of these conditions. The analysis demonstrates both similarities and differences between disease cohorts. Further investigations may elucidate varying subtypes of patients according to immune gene expression.

## Methods

### Sex as a biological variable.

Our study examined both male and female participants in the analysis. Sex was not considered as a covariate in this investigation, given the small sample size. A larger proportion of female participations were included in this investigation, given that females comprise the majority of people with ME/CFS (51).

### Participants.

All participants were recruited through the National Centre for NCNED participant database. People with ME/CFS were screened per the Fukuda criteria (52), CCC (53), and International Consensus Criteria (ICC) (54) using a comprehensive online questionnaire. Patients with ME/CFS were included if they fulfilled the CCC and/or ICC case definitions and reported being diagnosed by a physician. People with long COVID fulfilled the WHO working case definition for “Post COVID-19 Condition.” According to this definition, long COVID occurs in individuals with a history of probable or confirmed SARS-CoV-2 infection, usually 3 months from the onset of COVID-19, with symptoms that last for at least 2 months and cannot be explained by an alternative diagnosis (55). HC reported an absence of disease and/or chronic diagnoses.

In this study, symptoms were classified in 10 subtypes: (a) cognitive difficulties (e.g., cognitive overload, confusion, disorientation, impaired concentration, forgetfulness, and memory problems); (b) pain (e.g., headaches, muscle aches, and multijoint pain); (c) sleep disturbances (e.g., unrefreshing sleep, frequent awakenings, prolonged sleep, and reversed sleep cycle); (d) cardiovascular symptoms (e.g., orthostatic intolerance, cardiac arrhythmias, heart palpitations, lightheadedness, and dizziness); (e) respiratory symptoms (e.g., air hunger and difficulty breathing); (f) thermostatic intolerances (e.g., subnormal body temperature, abnormal sweating episodes, hot flushes, and cold extremities); (g) neurosensory or perceptual symptoms (e.g., inability to focus vision; impaired depth perception; sensitivity to touch, light, odor, taste, sound, and vibration; and poor balance or coordination); (8) urinary changes (e.g., changes to urination frequency and urgency to urinate); (h) immune disturbances (e.g., sore throat, tender lymph nodes, and new allergies/sensitivities); and (i) gastrointestinal disturbances (e.g., nausea, abdominal pain, bloating, diarrhea, and IBS).

All participants were aged between 18 and 65 years, did not report a BMI higher than 32.0 (kg/m^2^), and were nonsmokers. Participants were not included in this current study if they reported a history of alcohol abuse, cardiovascular disease, diabetes, metabolic syndrome, thyroid disease, malignancies, insomnia, anemia, or other fatigue-related illnesses or if they were pregnant or breastfeeding.

### Sample collection and preparation.

Between 20 and 40 mL of whole blood was collected from each participant into EDTA tubes via venipuncture by a qualified phlebotomist at collection locations including Griffith University (Gold Coast, Australia), Royal Brisbane and Women’s Hospital (Brisbane, Australia), Robina Hospital (Gold Coast, Australia), Toowoomba Base Hospital (Toowoomba, Australia), Sunshine Coast University Hospital (Sunshine Coast, Australia), and Tweed Hospital **(**Tweed, Australia**)**. EDTA whole blood (4 mL) was used for RBC count, WBC count, and granulocyte cell count within 4 hours of blood collection for each participant.

Samples were delivered to the laboratory deidentified using a unique code. Blood was used for peripheral blood mononuclear cells (PBMC) isolation by density gradient centrifugation at 350*g* using Ficoll (GE Healthcare) as previously described (56). PBMCs were stained with trypan blue (Invitrogen) to determine cell count and viability. In total, 1 × 10^7^ PBMCs were resuspended in FBS (Invitrogen) containing 10% dimethyl sulfoxide for storage at –80°C until RNA extraction.

Frozen PBMCs were thawed and immediately pelleted by centrifugation at 1,000*g*. Total RNA was isolated from PBMC pellets (5 × 10^6^ to 10 × 10^6^ cells) using either Trizol or an RNeasy Mini Kit (QIAGEN) according to manufacturer instructions. The concentration and quality of the RNA were checked using Nanodrop.

### RNA expression and NanoString.

Gene expression analysis of RNA was performed using the commercially available NanoString nCounter Immune Exhaustion gene expression panel (NanoString Technologies). This panel contains 785 genes to elucidate mechanisms behind T cell, B cell, and NK cell exhaustion in disease. A full list of investigated genes can be found in Supplemental Data 1. The quality of the samples was confirmed according to binding density, fields of view, positive controls, and negative controls. The range of binding density for all samples was within acceptable ranges.

Raw gene expression data were normalized against positive and negative controls to account for background noise and platform-associated variation. Normalization and analysis were performed using Rosalind Bio using geometric means of housekeeping genes (*ABCF1*, *ALAS1*, *EEF1G*, *G6PD*, *GAPDH*, *GUSB*, *HPRT1*, *OAZ1*, *POLR1B*, *POLR2A*, *PPIA*, *RPL19*, *SDHA*, *TBP*, *TUBB*) (Supplemental Data 1). Differential expression is reported between ME/CFS and long COVID with HC. IPA (Qiagen Digital Insights) was used for the interpretation of RNA in biological pathways and networks. The following filters were applied to all analyses: FC > 1.5 or < –1.5 and *P* < 0.05.

### Statistics.

Normality of participant data was determined using the Shapiro-Wilk test. Normally distributed continuous data was compared using 1-way ANOVA corrected using Bonferroni, and nonnormally distributed continuous data were compared using the Kruskal-Wallis test with Dunn’s corrections. Continuous data are presented as mean ± SD unless otherwise stated. Categorical variables were compared using the χ^2^ test and the Fisher’s exact test. Participant demographic data were analyzed using SPSS (version 27). Cell profile abundance scores were compared using 1-way ANOVA corrected using Bonferroni or the Kruskal-Wallis test with Dunn’s corrections using GraphPad Prism (version 10). Significance is set at *P* < 0.05. Adjusted *P* values (*P*_adj_) are provided unless otherwise stated.

### Study approval.

This project was approved by Griffith University Human Research Ethics Committee (GU:2022/666). All participants provided written consent prior to participation. Research involving human research participants was performed per the Declaration of Helsinki.

### Data availability.

The NanoString RNA-Seq data are available at National Centre for Biotechnology Information Gene Expression Omnibus database under accession no. GSE275334. Values for all data points in graphs are reported in the Supporting Data Values file.

## Author contributions

NEF, PR, L Herrero, and SMG conceptualized and design experimentation. NEF and PR isolated participant samples. NEF, PR, TE, L Herrero, and L Hool performed data analyses. SMG provided funding. All authors reviewed and approved the final manuscript.

## Supplementary Material

Supplemental data set 1

Supplemental data set 2

Supplemental data set 3

Supplemental data set 4

Supporting data values

## Figures and Tables

**Table 1:**
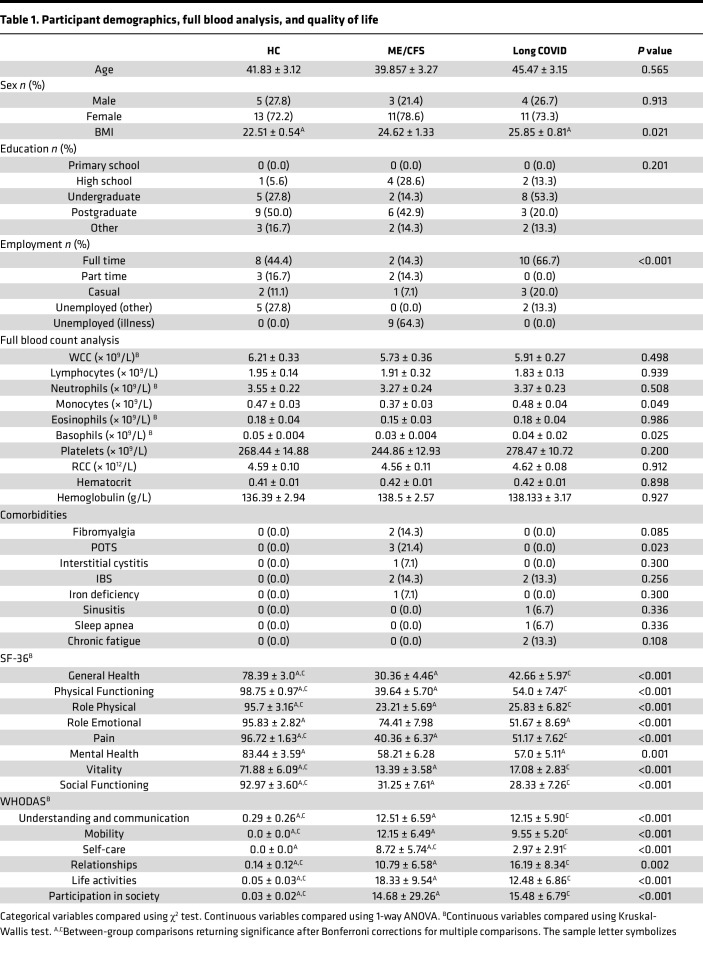
Participant demographics, full blood analysis, and quality of life

**Table 2:**
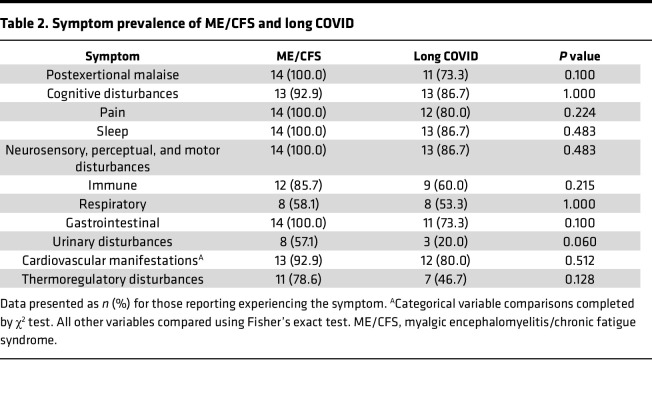
Symptom prevalence of ME/CFS and long COVID

**Table 3:**
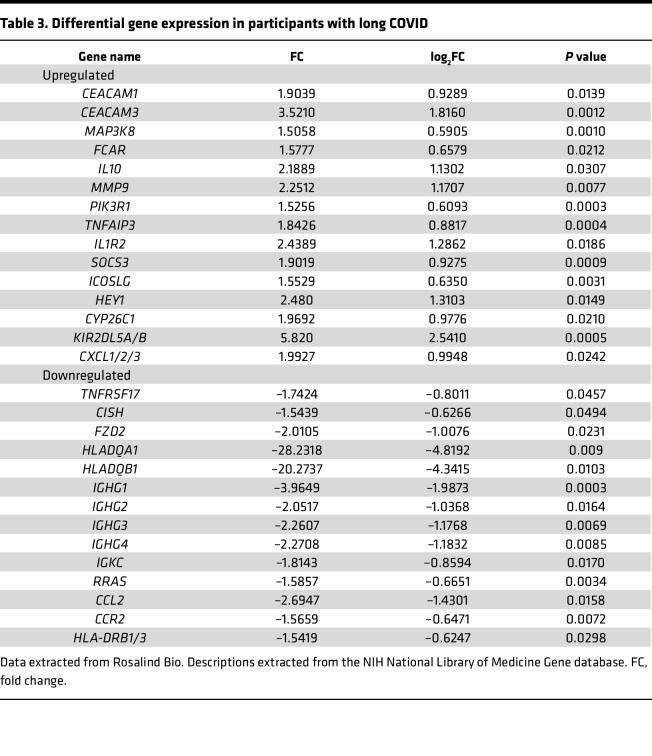
Differential gene expression in participants with long COVID

**Table 4:**
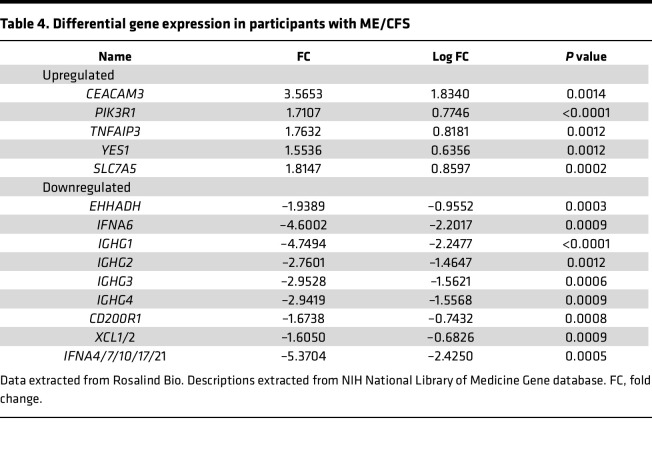
Differential gene expression in participants with ME/CFS

**Table 5:**
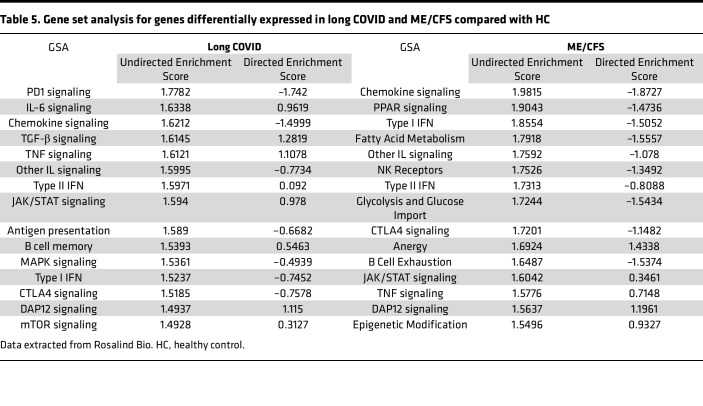
Gene set analysis for genes differentially expressed in long COVID and ME/CFS compared with HC

**Table 6:**
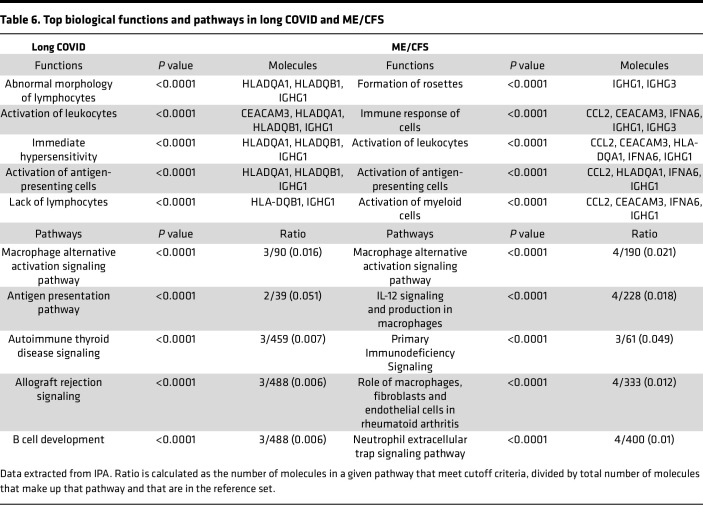
Top biological functions and pathways in long COVID and ME/CFS

## Version 1 10/22/2024

Electronic publication
